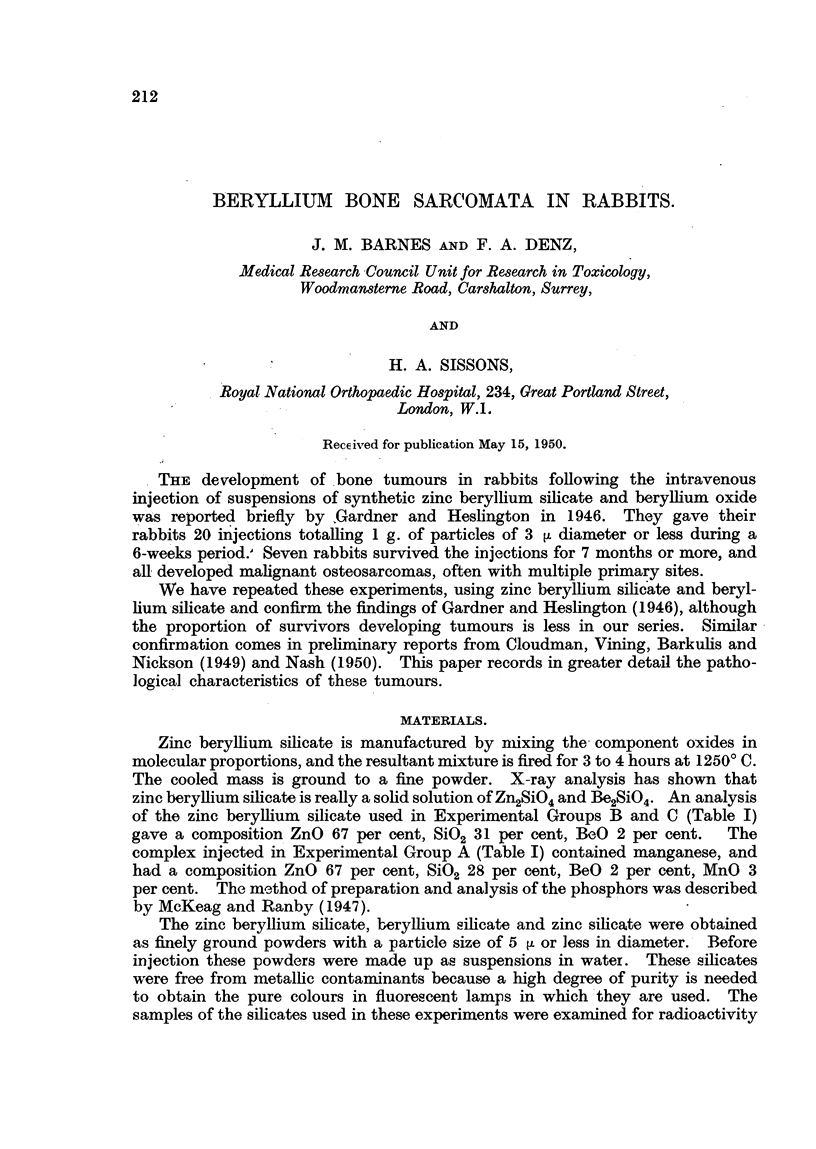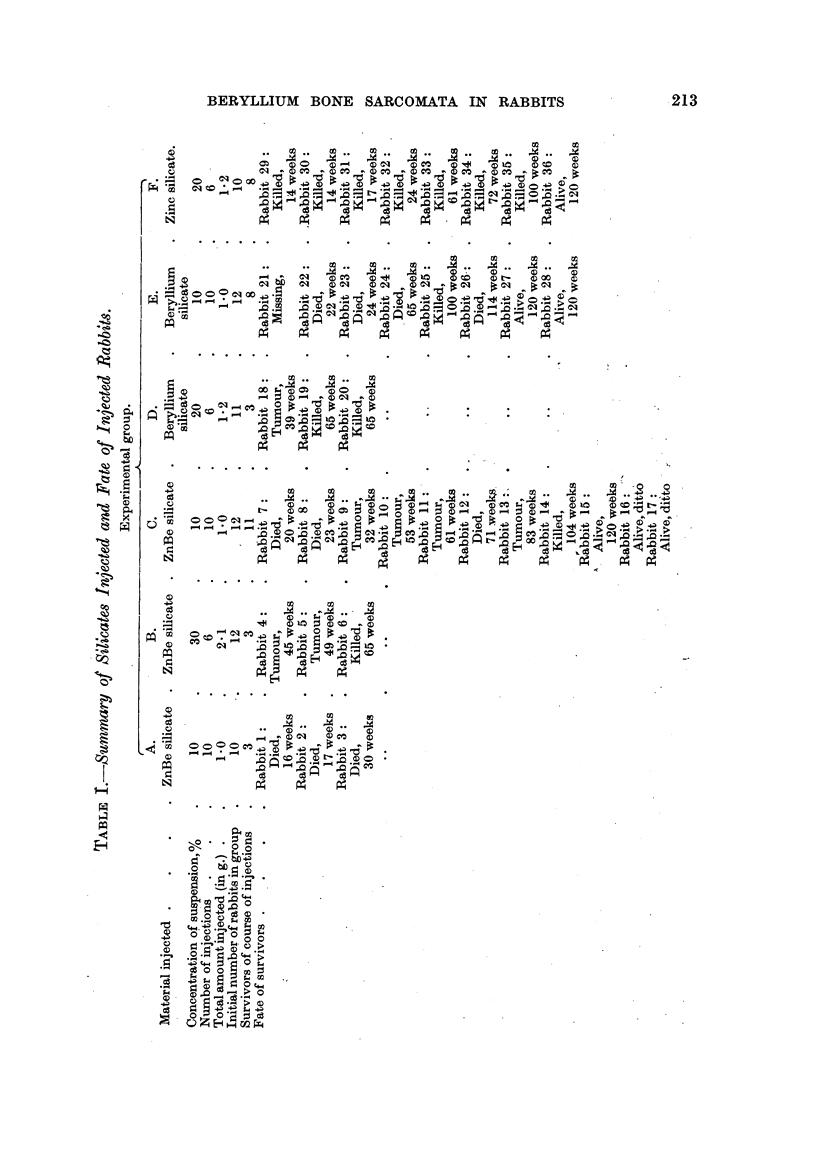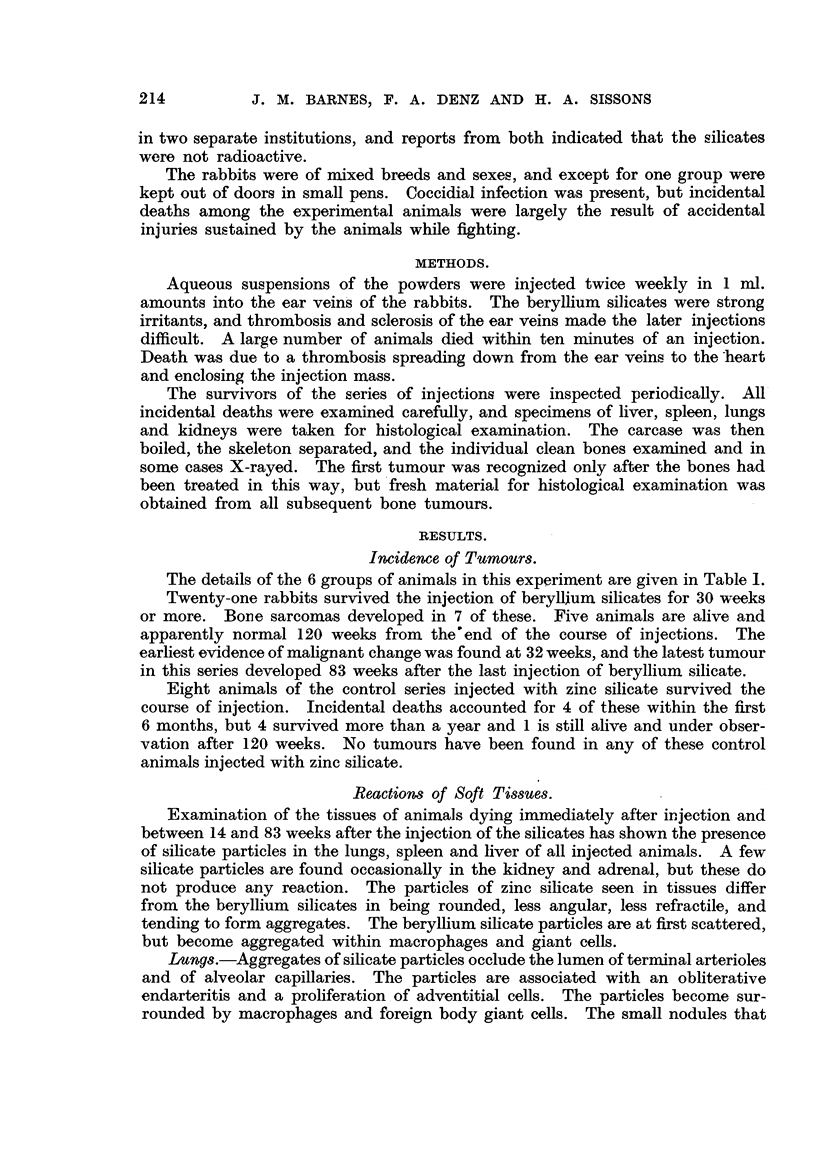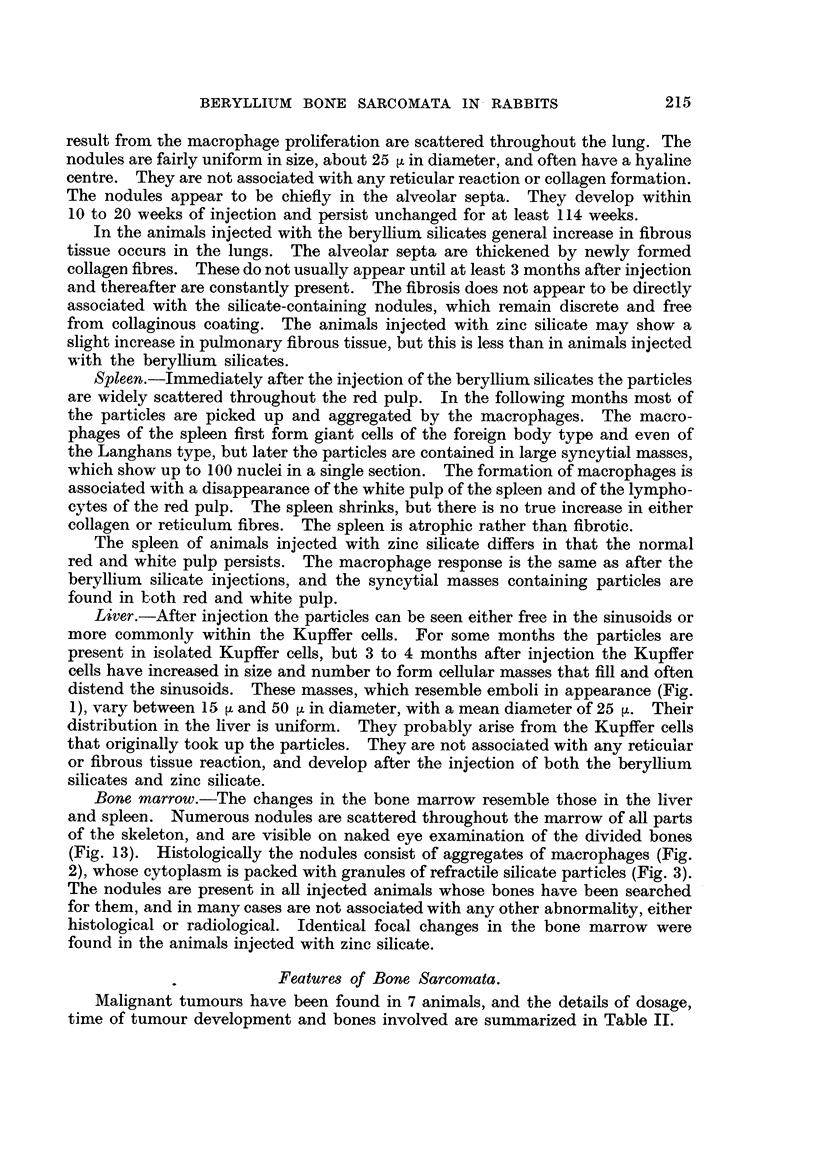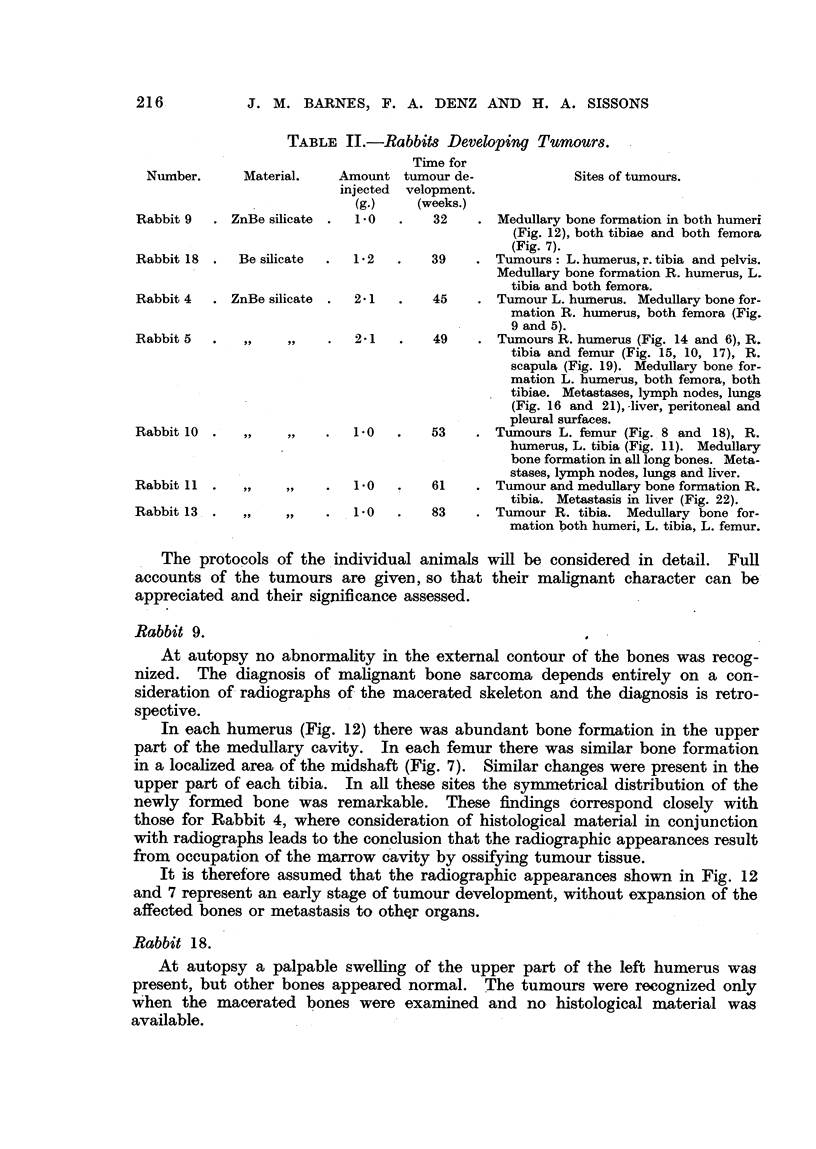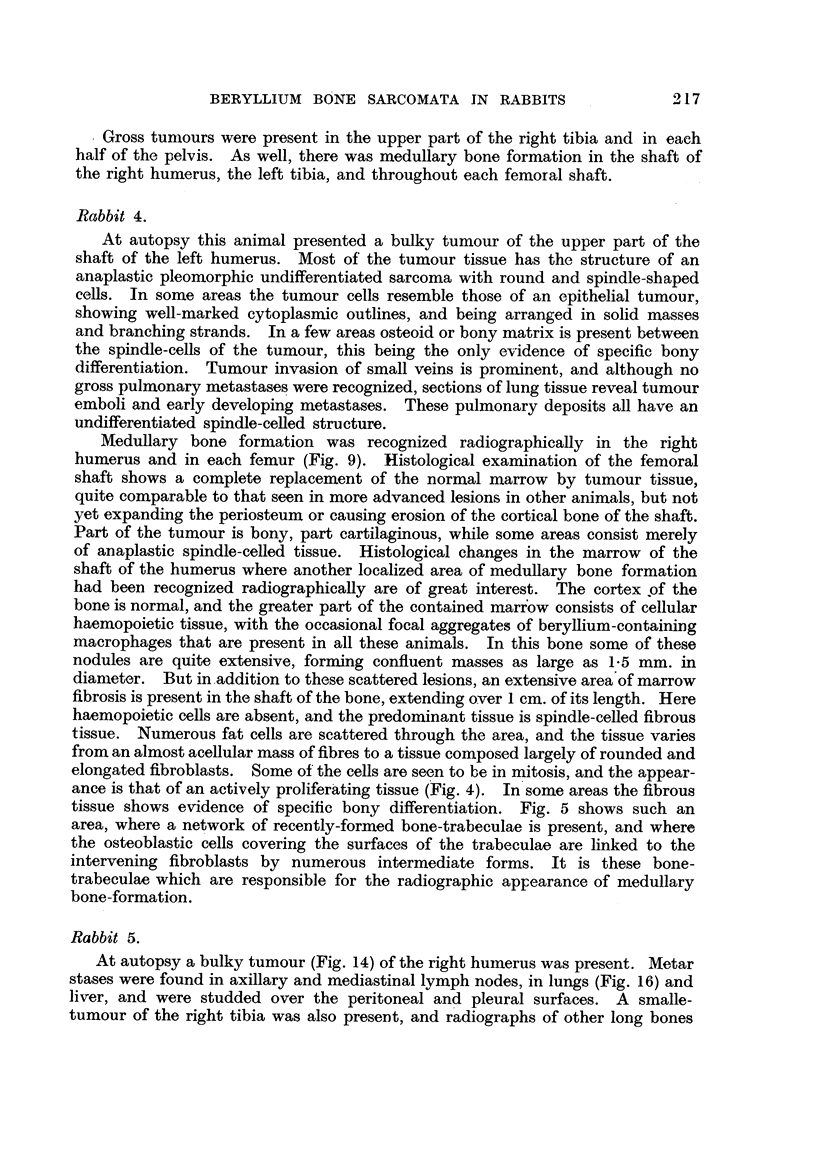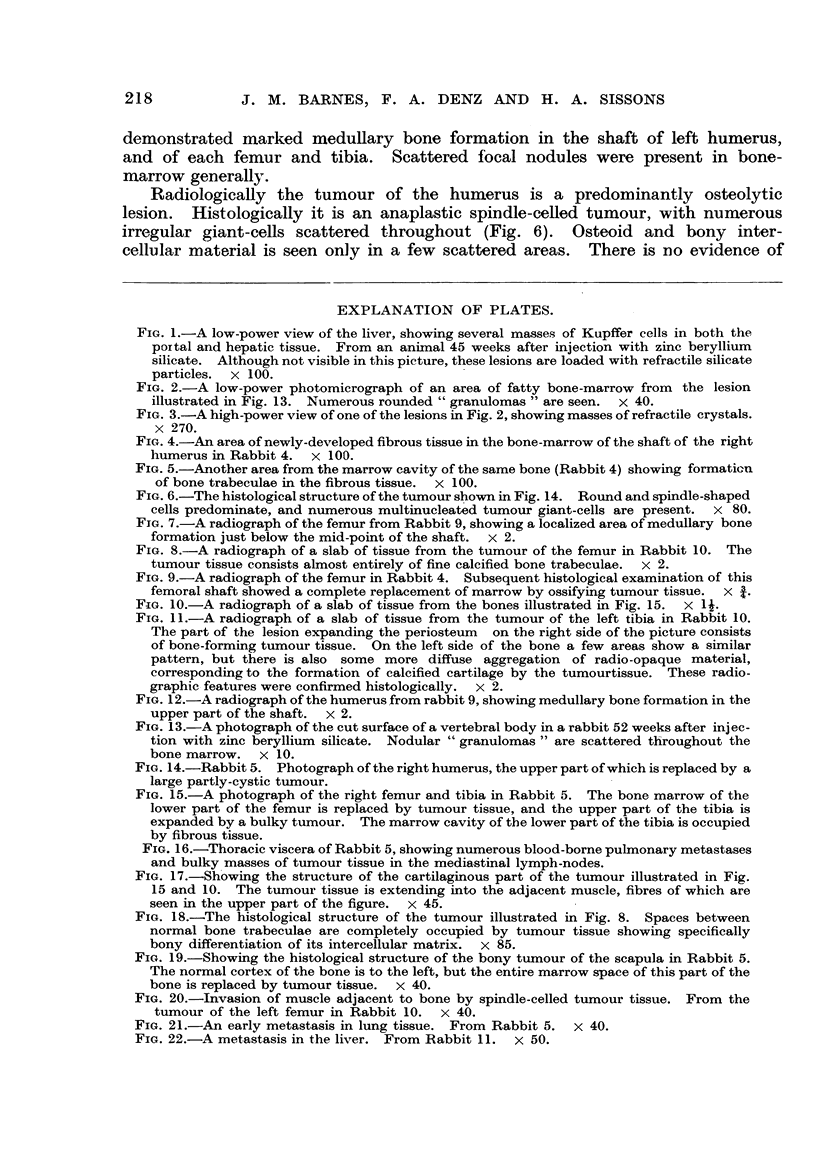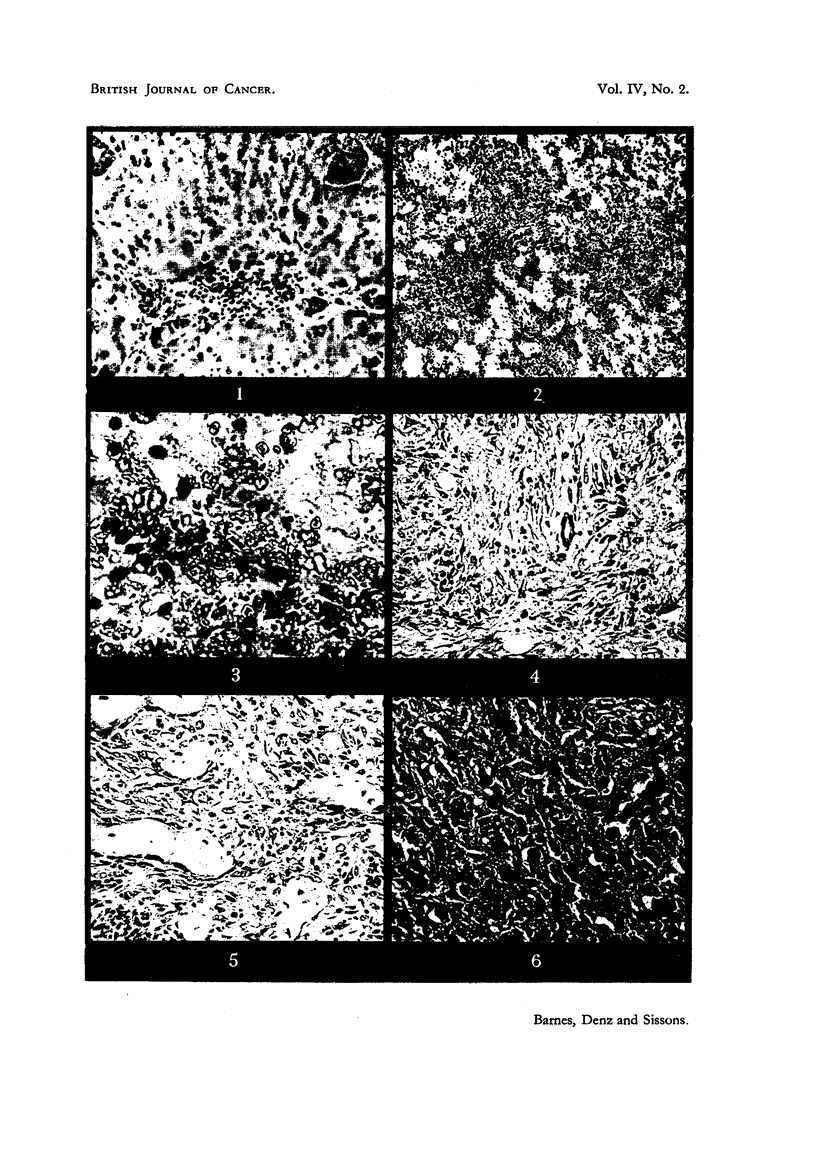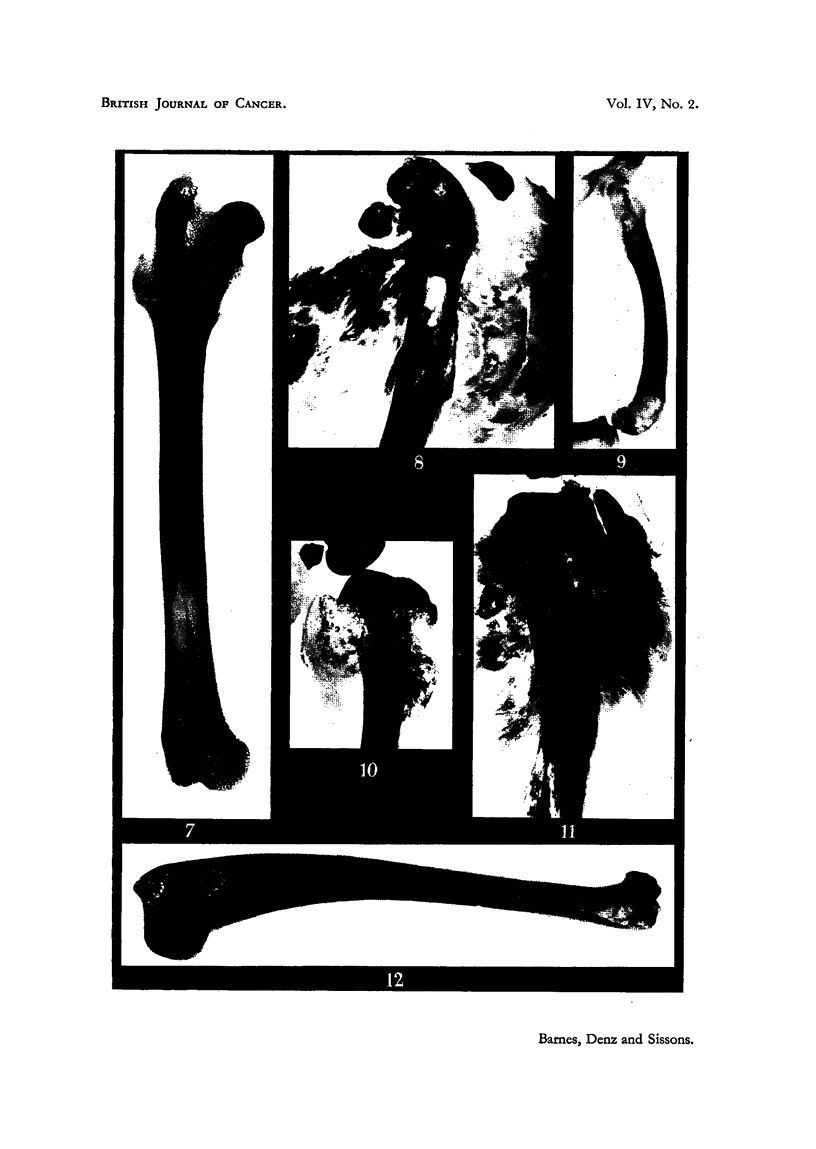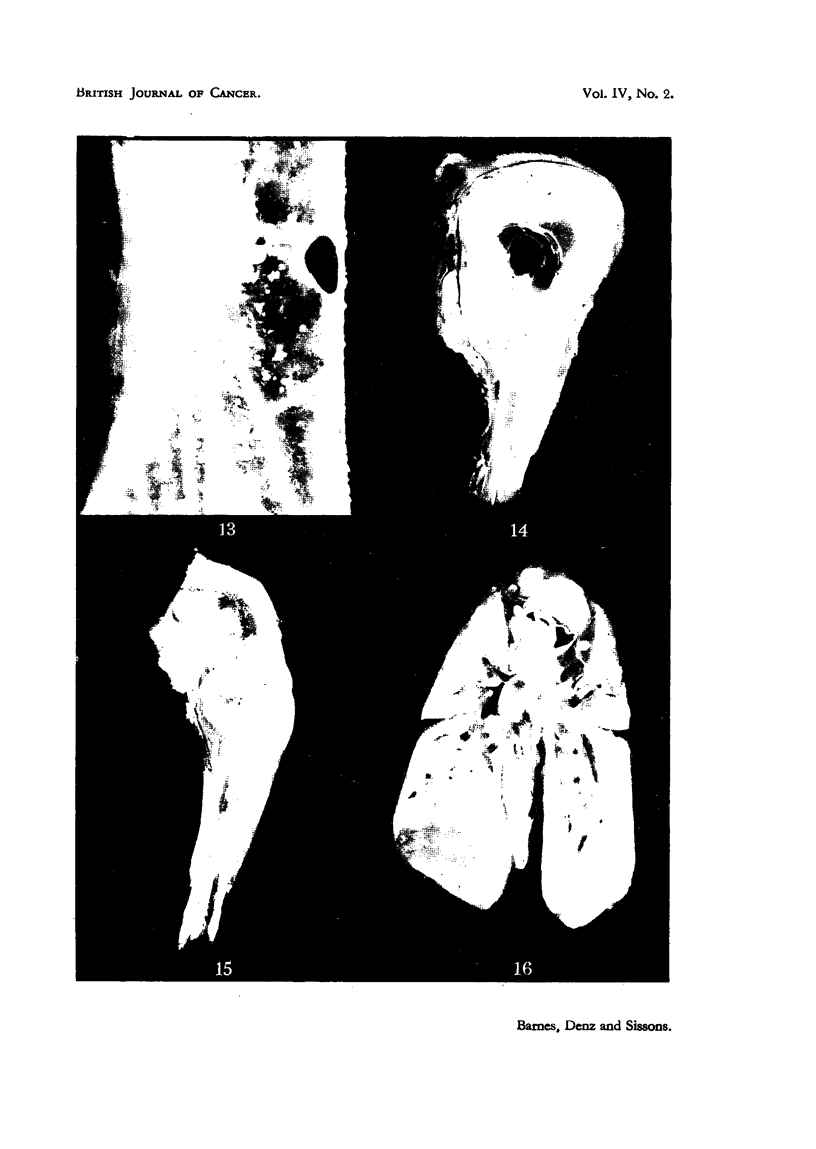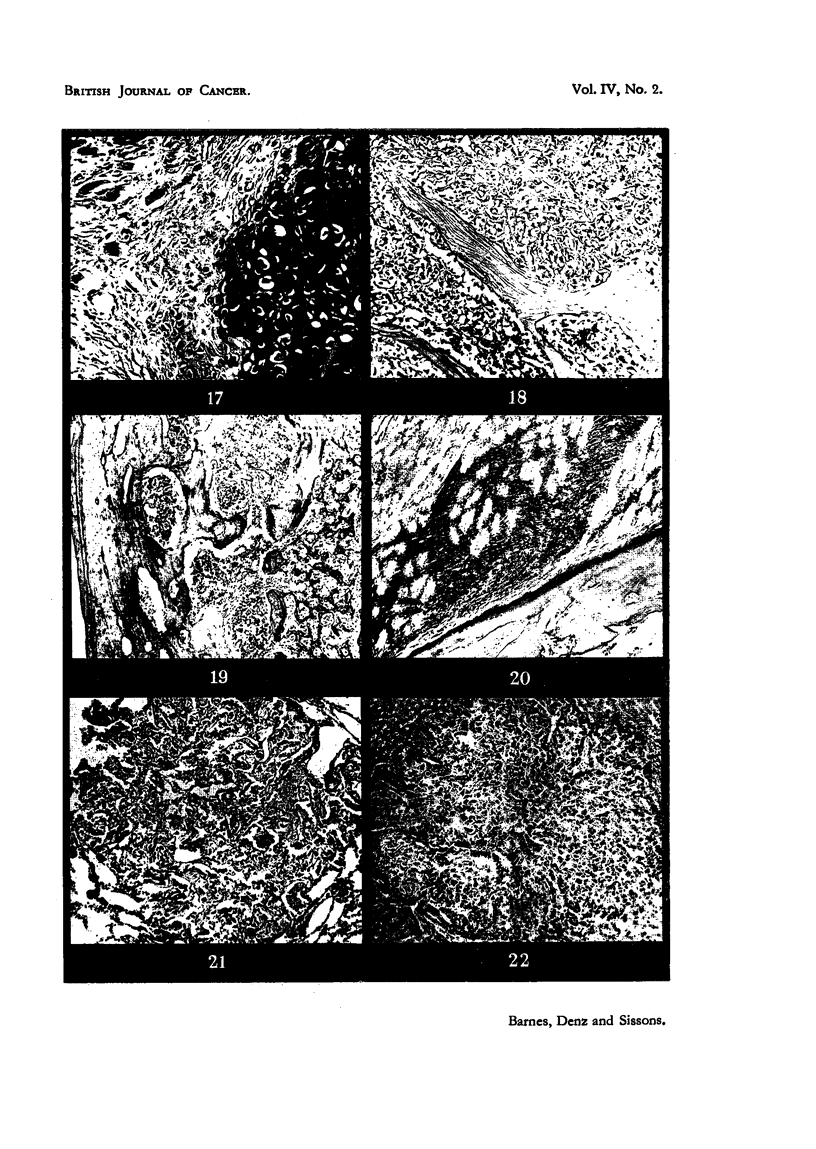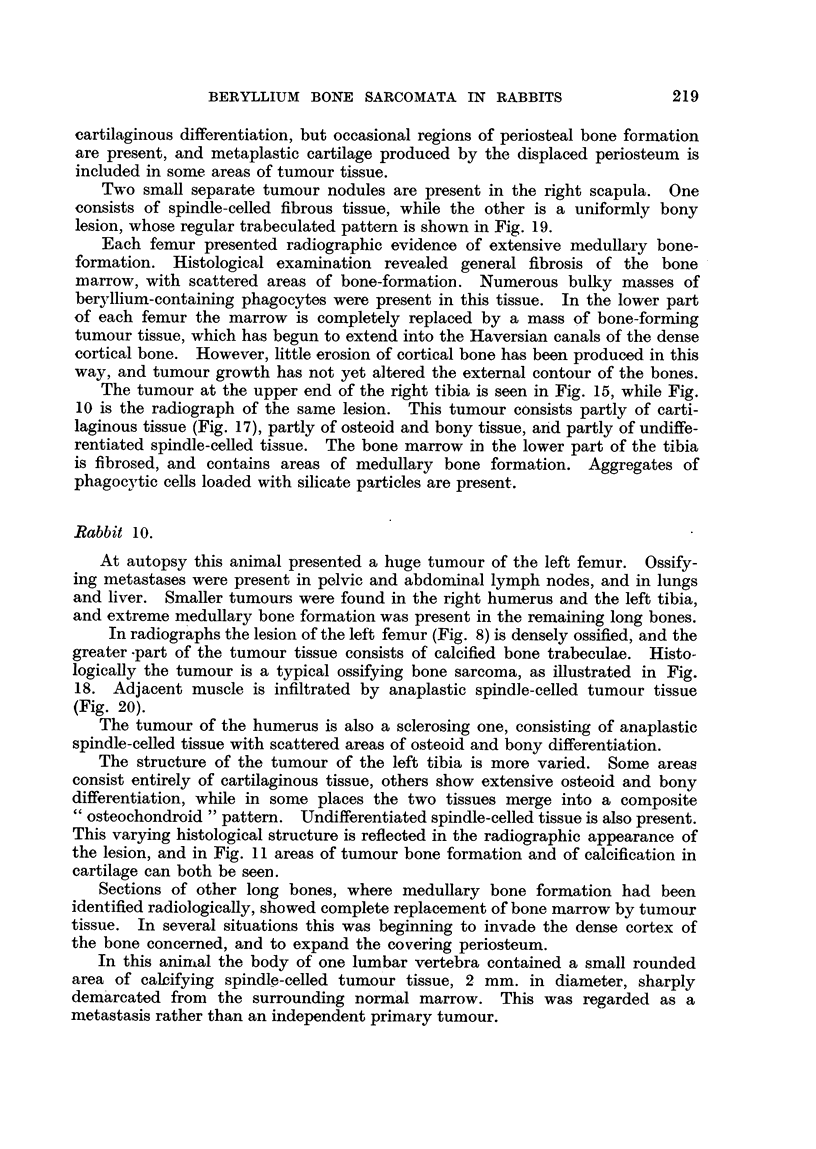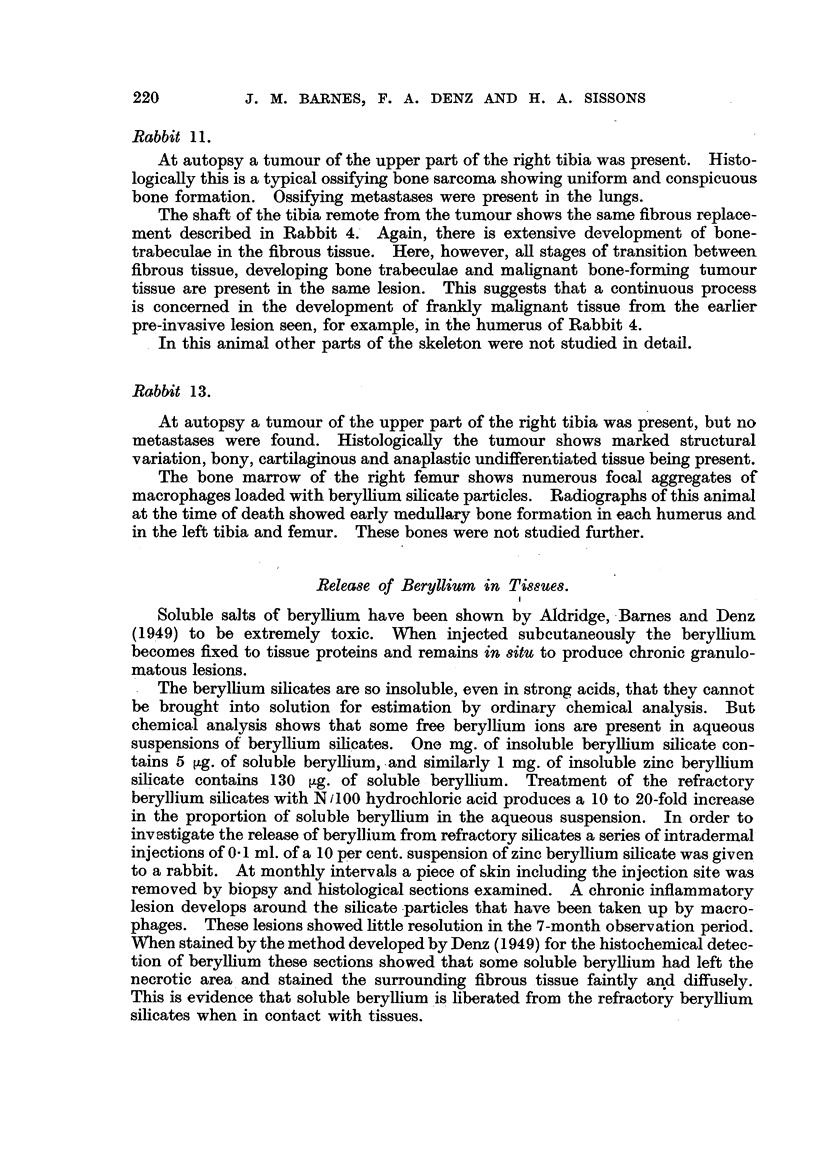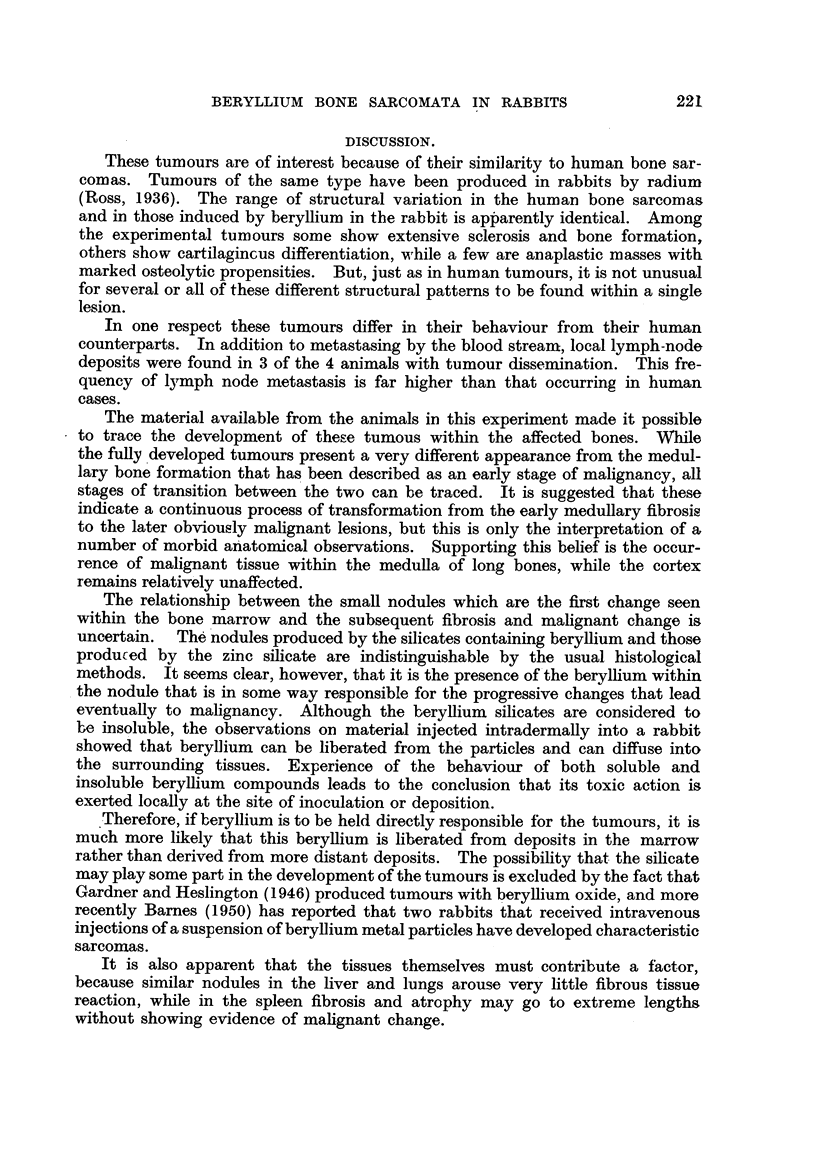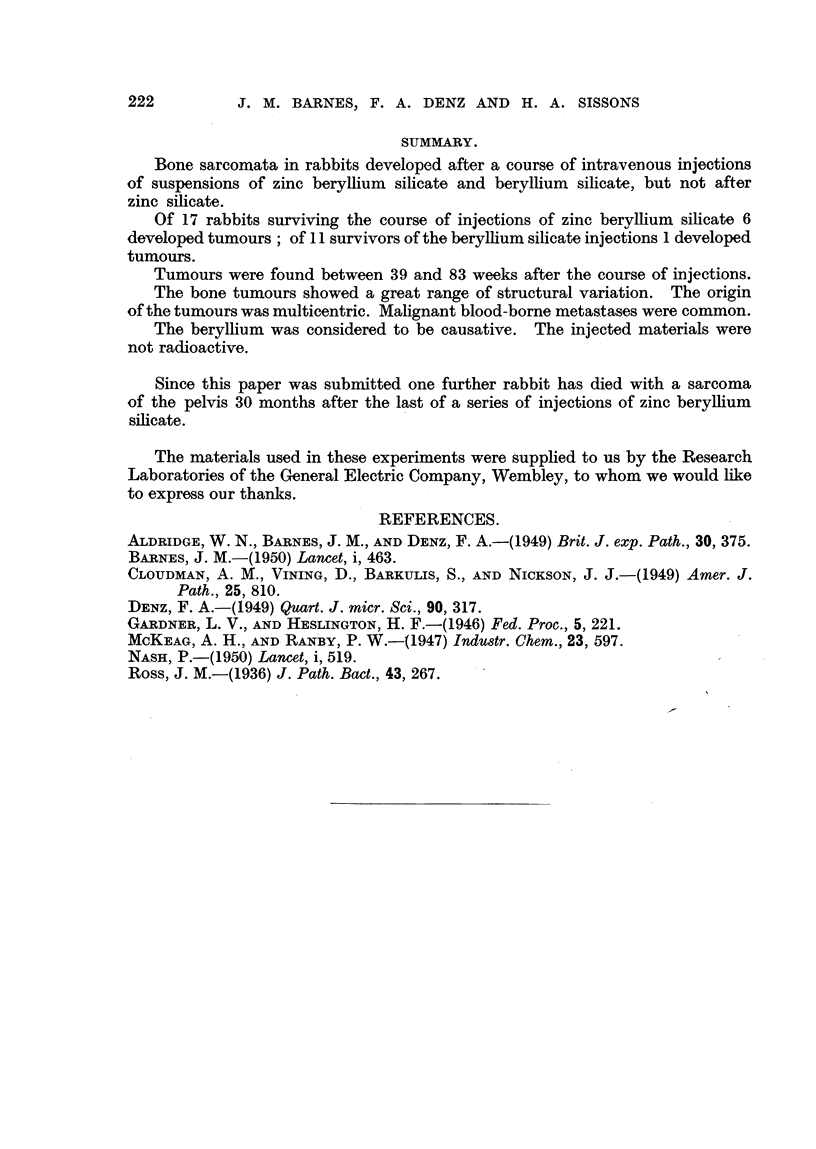# Beryllium Bone Sarcomata in Rabbits

**DOI:** 10.1038/bjc.1950.21

**Published:** 1950-06

**Authors:** J. M. Barnes, F. A. Denz, H. A. Sissons

## Abstract

**Images:**


### BERYLLIUM BONE SARCOMATA IN RABBITS.

J. M. BARNES AND F. A. DENZ,

*Medical Research Council Unit for Research in Toxicology, Woodmansterne Road, Carshalton, Surrey,*

AND

H. A. SISSONS,

*Royal National Orthopaedic Hospital, 234, Great Portland Street, London, W.1.*

Received for publication May 15, 1950.

THE development of bone tumours in rabbits following the intravenous injection of suspensions of synthetic zinc beryllium silicate and beryllium oxide was reported briefly by Gardner and Heslington in 1946. They gave their rabbits 20 injections totalling 1 g. of particles of 3 μ diameter or less during a 6-weeks period. Seven rabbits survived the injections for 7 months or more, and all developed malignant osteosarcomas, often with multiple primary sites.

We have repeated these experiments, using zinc beryllium silicate and beryllium silicate and confirm the findings of Gardner and Heslington (1946), although the proportion of survivors developing tumours is less in our series. Similar confirmation comes in preliminary reports from Cloudman, Vining, Barkulis and Nickson (1949) and Nash (1950). This paper records in greater detail the pathological characteristics of these tumours.

### MATERIALS.

Zinc beryllium silicate is manufactured by mixing the component oxides in molecular proportions, and the resultant mixture is fired for 3 to 4 hours at 1250° C. The cooled mass is ground to a fine powder. X-ray analysis has shown that zinc beryllium silicate is really a solid solution of $Zn_2SiO_4$ and $Be_2SiO_4$. An analysis of the zinc beryllium silicate used in Experimental Groups B and C (Table I) gave a composition ZnO 67 per cent, $SiO_2$ 31 per cent, BeO 2 per cent. The complex injected in Experimental Group A (Table I) contained manganese, and had a composition ZnO 67 per cent, $SiO_2$ 28 per cent, BeO 2 per cent, MnO 3 per cent. The method of preparation and analysis of the phosphors was described by McKeag and Ranby (1947).

The zinc beryllium silicate, beryllium silicate and zinc silicate were obtained as finely ground powders with a particle size of 5 μ or less in diameter. Before injection these powders were made up as suspensions in water. These silicates were free from metallic contaminants because a high degree of purity is needed to obtain the pure colours in fluorescent lamps in which they are used. The samples of the silicates used in these experiments were examined for radioactivity

TABLE I.—*Summary of Silicates Injected and Fate of Injected Rabbits.*

| | Experimental group. | | | | | |
|---|---|---|---|---|---|---|
| | A. | B. | C. | D. | E. | F. |
| Material injected | ZnBe silicate | ZnBe silicate | ZnBe silicate | Beryllium silicate | Beryllium silicate | Zinc silicate. |
| Concentration of suspension, % | 10 | 30 | 10 | 20 | 10 | 20 |
| Number of injections | 10 | 6 | 10 | 6 | 10 | 6 |
| Total amount injected (in g.) | 1·0 | 2·1 | 1·0 | 1·2 | 1·0 | 1·2 |
| Initial number of rabbits in group | 10 | 12 | 12 | 11 | 12 | 10 |
| Survivors of course of injections | 3 | 3 | 11 | 3 | 8 | 8 |
| Fate of survivors | Rabbit 1 : Died, 16 weeks | Rabbit 4 : Tumour, 45 weeks | Rabbit 7 : Died, 20 weeks | Rabbit 18 : Tumour, 39 weeks | Rabbit 21 : Missing, | Rabbit 29 : Killed, 14 weeks |
| | Rabbit 2 : Died, 17 weeks | Rabbit 5 : Tumour, 49 weeks | Rabbit 8 : Died, 23 weeks | Rabbit 19 : Killed, 65 weeks | Rabbit 22 : Died, 22 weeks | Rabbit 30 : Killed, 14 weeks |
| | Rabbit 3 : Died, 30 weeks | Rabbit 6 : Killed, 65 weeks | Rabbit 9 : Tumour, 32 weeks | Rabbit 20 : Killed, 65 weeks | Rabbit 23 : Died, 24 weeks | Rabbit 31 : Killed, 17 weeks |
| | .. | .. | Rabbit 10 : Tumour, 53 weeks | .. | Rabbit 24 : Died, 65 weeks | Rabbit 32 : Killed, 24 weeks |
| | | | Rabbit 11 : Tumour, 61 weeks | .. | Rabbit 25 : Killed, 100 weeks | Rabbit 33 : Killed, 61 weeks |
| | | | Rabbit 12 : Died, 71 weeks | .. | Rabbit 26 : Died, 114 weeks | Rabbit 34 : Killed, 72 weeks |
| | | | Rabbit 13 : Tumour, 83 weeks | .. | Rabbit 27 : Alive, 120 weeks | Rabbit 35 : Killed, 100 weeks |
| | | | Rabbit 14 : Killed, 104 weeks | .. | Rabbit 28 : Alive, 120 weeks | Rabbit 36 : Alive, 120 weeks |
| | | | Rabbit 15 : Alive, 120 weeks | | | |
| | | | Rabbit 16 : Alive, ditto | | | |
| | | | Rabbit 17 : Alive, ditto | | | |

in two separate institutions, and reports from both indicated that the silicates were not radioactive.

The rabbits were of mixed breeds and sexes, and except for one group were kept out of doors in small pens. Coccidial infection was present, but incidental deaths among the experimental animals were largely the result of accidental injuries sustained by the animals while fighting.

### METHODS.

Aqueous suspensions of the powders were injected twice weekly in 1 ml. amounts into the ear veins of the rabbits. The beryllium silicates were strong irritants, and thrombosis and sclerosis of the ear veins made the later injections difficult. A large number of animals died within ten minutes of an injection. Death was due to a thrombosis spreading down from the ear veins to the heart and enclosing the injection mass.

The survivors of the series of injections were inspected periodically. All incidental deaths were examined carefully, and specimens of liver, spleen, lungs and kidneys were taken for histological examination. The carcase was then boiled, the skeleton separated, and the individual clean bones examined and in some cases X-rayed. The first tumour was recognized only after the bones had been treated in this way, but fresh material for histological examination was obtained from all subsequent bone tumours.

### RESULTS.

### *Incidence of Tumours.*

The details of the 6 groups of animals in this experiment are given in Table I.

Twenty-one rabbits survived the injection of beryllium silicates for 30 weeks or more. Bone sarcomas developed in 7 of these. Five animals are alive and apparently normal 120 weeks from the end of the course of injections. The earliest evidence of malignant change was found at 32 weeks, and the latest tumour in this series developed 83 weeks after the last injection of beryllium silicate.

Eight animals of the control series injected with zinc silicate survived the course of injection. Incidental deaths accounted for 4 of these within the first 6 months, but 4 survived more than a year and 1 is still alive and under observation after 120 weeks. No tumours have been found in any of these control animals injected with zinc silicate.

### *Reactions of Soft Tissues.*

Examination of the tissues of animals dying immediately after injection and between 14 and 83 weeks after the injection of the silicates has shown the presence of silicate particles in the lungs, spleen and liver of all injected animals. A few silicate particles are found occasionally in the kidney and adrenal, but these do not produce any reaction. The particles of zinc silicate seen in tissues differ from the beryllium silicates in being rounded, less angular, less refractile, and tending to form aggregates. The beryllium silicate particles are at first scattered, but become aggregated within macrophages and giant cells.

*Lungs.*—Aggregates of silicate particles occlude the lumen of terminal arterioles and of alveolar capillaries. The particles are associated with an obliterative endarteritis and a proliferation of adventitial cells. The particles become surrounded by macrophages and foreign body giant cells. The small nodules that result from the macrophage proliferation are scattered throughout the lung. The nodules are fairly uniform in size, about 25 μ in diameter, and often have a hyaline centre. They are not associated with any reticular reaction or collagen formation. The nodules appear to be chiefly in the alveolar septa. They develop within 10 to 20 weeks of injection and persist unchanged for at least 114 weeks.

In the animals injected with the beryllium silicates general increase in fibrous tissue occurs in the lungs. The alveolar septa are thickened by newly formed collagen fibres. These do not usually appear until at least 3 months after injection and thereafter are constantly present. The fibrosis does not appear to be directly associated with the silicate-containing nodules, which remain discrete and free from collaginous coating. The animals injected with zinc silicate may show a slight increase in pulmonary fibrous tissue, but this is less than in animals injected with the beryllium silicates.

*Spleen.*—Immediately after the injection of the beryllium silicates the particles are widely scattered throughout the red pulp. In the following months most of the particles are picked up and aggregated by the macrophages. The macrophages of the spleen first form giant cells of the foreign body type and even of the Langhans type, but later the particles are contained in large syncytial masses, which show up to 100 nuclei in a single section. The formation of macrophages is associated with a disappearance of the white pulp of the spleen and of the lymphocytes of the red pulp. The spleen shrinks, but there is no true increase in either collagen or reticulum fibres. The spleen is atrophic rather than fibrotic.

The spleen of animals injected with zinc silicate differs in that the normal red and white pulp persists. The macrophage response is the same as after the beryllium silicate injections, and the syncytial masses containing particles are found in both red and white pulp.

*Liver.*—After injection the particles can be seen either free in the sinusoids or more commonly within the Kupffer cells. For some months the particles are present in isolated Kupffer cells, but 3 to 4 months after injection the Kupffer cells have increased in size and number to form cellular masses that fill and often distend the sinusoids. These masses, which resemble emboli in appearance (Fig. 1), vary between 15 μ and 50 μ in diameter, with a mean diameter of 25 μ. Their distribution in the liver is uniform. They probably arise from the Kupffer cells that originally took up the particles. They are not associated with any reticular or fibrous tissue reaction, and develop after the injection of both the beryllium silicates and zinc silicate.

*Bone marrow.*—The changes in the bone marrow resemble those in the liver and spleen. Numerous nodules are scattered throughout the marrow of all parts of the skeleton, and are visible on naked eye examination of the divided bones (Fig. 13). Histologically the nodules consist of aggregates of macrophages (Fig. 2), whose cytoplasm is packed with granules of refractile silicate particles (Fig. 3). The nodules are present in all injected animals whose bones have been searched for them, and in many cases are not associated with any other abnormality, either histological or radiological. Identical focal changes in the bone marrow were found in the animals injected with zinc silicate.

### *Features of Bone Sarcomata.*

Malignant tumours have been found in 7 animals, and the details of dosage, time of tumour development and bones involved are summarized in Table II.

TABLE II.—*Rabbits Developing Tumours.*

| Number. | Material. | Amount injected (g.) | Time for tumour development. (weeks.) | Sites of tumours. |
|---|---|---|---|---|
| Rabbit 9 | ZnBe silicate | 1·0 | 32 | Medullary bone formation in both humeri (Fig. 12), both tibiae and both femora (Fig. 7). |
| Rabbit 18 | Be silicate | 1·2 | 39 | Tumours : L. humerus, r. tibia and pelvis. Medullary bone formation R. humerus, L. tibia and both femora. |
| Rabbit 4 | ZnBe silicate | 2·1 | 45 | Tumour L. humerus. Medullary bone formation R. humerus, both femora (Fig. 9 and 5). |
| Rabbit 5 | ,, ,, | 2·1 | 49 | Tumours R. humerus (Fig. 14 and 6), R. tibia and femur (Fig. 15, 10, 17), R. scapula (Fig. 19). Medullary bone formation L. humerus, both femora, both tibiae. Metastases, lymph nodes, lungs (Fig. 16 and 21), liver, peritoneal and pleural surfaces. |
| Rabbit 10 | ,, ,, | 1·0 | 53 | Tumours L. femur (Fig. 8 and 18), R. humerus, L. tibia (Fig. 11). Medullary bone formation in all long bones. Metastases, lymph nodes, lungs and liver. |
| Rabbit 11 | ,, ,, | 1·0 | 61 | Tumour and medullary bone formation R. tibia. Metastasis in liver (Fig. 22). |
| Rabbit 13 | ,, ,, | 1·0 | 83 | Tumour R. tibia. Medullary bone formation both humeri, L. tibia, L. femur. |

The protocols of the individual animals will be considered in detail. Full accounts of the tumours are given, so that their malignant character can be appreciated and their significance assessed.

*Rabbit* 9.

At autopsy no abnormality in the external contour of the bones was recognized. The diagnosis of malignant bone sarcoma depends entirely on a consideration of radiographs of the macerated skeleton and the diagnosis is retrospective.

In each humerus (Fig. 12) there was abundant bone formation in the upper part of the medullary cavity. In each femur there was similar bone formation in a localized area of the midshaft (Fig. 7). Similar changes were present in the upper part of each tibia. In all these sites the symmetrical distribution of the newly formed bone was remarkable. These findings correspond closely with those for Rabbit 4, where consideration of histological material in conjunction with radiographs leads to the conclusion that the radiographic appearances result from occupation of the marrow cavity by ossifying tumour tissue.

It is therefore assumed that the radiographic appearances shown in Fig. 12 and 7 represent an early stage of tumour development, without expansion of the affected bones or metastasis to other organs.

*Rabbit* 18.

At autopsy a palpable swelling of the upper part of the left humerus was present, but other bones appeared normal. The tumours were recognized only when the macerated bones were examined and no histological material was available.

Gross tumours were present in the upper part of the right tibia and in each half of the pelvis. As well, there was medullary bone formation in the shaft of the right humerus, the left tibia, and throughout each femoral shaft.

*Rabbit* 4.

At autopsy this animal presented a bulky tumour of the upper part of the shaft of the left humerus. Most of the tumour tissue has the structure of an anaplastic pleomorphic undifferentiated sarcoma with round and spindle-shaped cells. In some areas the tumour cells resemble those of an epithelial tumour, showing well-marked cytoplasmic outlines, and being arranged in solid masses and branching strands. In a few areas osteoid or bony matrix is present between the spindle-cells of the tumour, this being the only evidence of specific bony differentiation. Tumour invasion of small veins is prominent, and although no gross pulmonary metastases were recognized, sections of lung tissue reveal tumour emboli and early developing metastases. These pulmonary deposits all have an undifferentiated spindle-celled structure.

Medullary bone formation was recognized radiographically in the right humerus and in each femur (Fig. 9). Histological examination of the femoral shaft shows a complete replacement of the normal marrow by tumour tissue, quite comparable to that seen in more advanced lesions in other animals, but not yet expanding the periosteum or causing erosion of the cortical bone of the shaft. Part of the tumour is bony, part cartilaginous, while some areas consist merely of anaplastic spindle-celled tissue. Histological changes in the marrow of the shaft of the humerus where another localized area of medullary bone formation had been recognized radiographically are of great interest. The cortex of the bone is normal, and the greater part of the contained marrow consists of cellular haemopoietic tissue, with the occasional focal aggregates of beryllium-containing macrophages that are present in all these animals. In this bone some of these nodules are quite extensive, forming confluent masses as large as 1·5 mm. in diameter. But in addition to these scattered lesions, an extensive area of marrow fibrosis is present in the shaft of the bone, extending over 1 cm. of its length. Here haemopoietic cells are absent, and the predominant tissue is spindle-celled fibrous tissue. Numerous fat cells are scattered through the area, and the tissue varies from an almost acellular mass of fibres to a tissue composed largely of rounded and elongated fibroblasts. Some of the cells are seen to be in mitosis, and the appearance is that of an actively proliferating tissue (Fig. 4). In some areas the fibrous tissue shows evidence of specific bony differentiation. Fig. 5 shows such an area, where a network of recently-formed bone-trabeculae is present, and where the osteoblastic cells covering the surfaces of the trabeculae are linked to the intervening fibroblasts by numerous intermediate forms. It is these bone-trabeculae which are responsible for the radiographic appearance of medullary bone-formation.

*Rabbit* 5.

At autopsy a bulky tumour (Fig. 14) of the right humerus was present. Metar stases were found in axillary and mediastinal lymph nodes, in lungs (Fig. 16) and liver, and were studded over the peritoneal and pleural surfaces. A smalle-tumour of the right tibia was also present, and radiographs of other long bones demonstrated marked medullary bone formation in the shaft of left humerus, and of each femur and tibia. Scattered focal nodules were present in bone-marrow generally.

Radiologically the tumour of the humerus is a predominantly osteolytic lesion. Histologically it is an anaplastic spindle-celled tumour, with numerous irregular giant-cells scattered throughout (Fig. 6). Osteoid and bony intercellular material is seen only in a few scattered areas. There is no evidence of

---

EXPLANATION OF PLATES.

FIG. 1.—A low-power view of the liver, showing several masses of Kupffer cells in both the portal and hepatic tissue. From an animal 45 weeks after injection with zinc beryllium silicate. Although not visible in this picture, these lesions are loaded with refractile silicate particles. × 100.

FIG. 2.—A low-power photomicrograph of an area of fatty bone-marrow from the lesion illustrated in Fig. 13. Numerous rounded " granulomas " are seen. × 40.

FIG. 3.—A high-power view of one of the lesions in Fig. 2, showing masses of refractile crystals. × 270.

FIG. 4.—An area of newly-developed fibrous tissue in the bone-marrow of the shaft of the right humerus in Rabbit 4. × 100.

FIG. 5.—Another area from the marrow cavity of the same bone (Rabbit 4) showing formation of bone trabeculae in the fibrous tissue. × 100.

FIG. 6.—The histological structure of the tumour shown in Fig. 14. Round and spindle-shaped cells predominate, and numerous multinucleated tumour giant-cells are present. × 80.

FIG. 7.—A radiograph of the femur from Rabbit 9, showing a localized area of medullary bone formation just below the mid-point of the shaft. × 2.

FIG. 8.—A radiograph of a slab of tissue from the tumour of the femur in Rabbit 10. The tumour tissue consists almost entirely of fine calcified bone trabeculae. × 2.

FIG. 9.—A radiograph of the femur in Rabbit 4. Subsequent histological examination of this femoral shaft showed a complete replacement of marrow by ossifying tumour tissue. × $\frac{3}{4}$.

FIG. 10.—A radiograph of a slab of tissue from the bones illustrated in Fig. 15. × $1\frac{1}{2}$.

FIG. 11.—A radiograph of a slab of tissue from the tumour of the left tibia in Rabbit 10. The part of the lesion expanding the periosteum on the right side of the picture consists of bone-forming tumour tissue. On the left side of the bone a few areas show a similar pattern, but there is also some more diffuse aggregation of radio-opaque material, corresponding to the formation of calcified cartilage by the tumourtissue. These radiographic features were confirmed histologically. × 2.

FIG. 12.—A radiograph of the humerus from rabbit 9, showing medullary bone formation in the upper part of the shaft. × 2.

FIG. 13.—A photograph of the cut surface of a vertebral body in a rabbit 52 weeks after injection with zinc beryllium silicate. Nodular " granulomas " are scattered throughout the bone marrow. × 10.

FIG. 14.—Rabbit 5. Photograph of the right humerus, the upper part of which is replaced by a large partly-cystic tumour.

FIG. 15.—A photograph of the right femur and tibia in Rabbit 5. The bone marrow of the lower part of the femur is replaced by tumour tissue, and the upper part of the tibia is expanded by a bulky tumour. The marrow cavity of the lower part of the tibia is occupied by fibrous tissue.

FIG. 16.—Thoracic viscera of Rabbit 5, showing numerous blood-borne pulmonary metastases and bulky masses of tumour tissue in the mediastinal lymph-nodes.

FIG. 17.—Showing the structure of the cartilaginous part of the tumour illustrated in Fig. 15 and 10. The tumour tissue is extending into the adjacent muscle, fibres of which are seen in the upper part of the figure. × 45.

FIG. 18.—The histological structure of the tumour illustrated in Fig. 8. Spaces between normal bone trabeculae are completely occupied by tumour tissue showing specifically bony differentiation of its intercellular matrix. × 85.

FIG. 19.—Showing the histological structure of the bony tumour of the scapula in Rabbit 5. The normal cortex of the bone is to the left, but the entire marrow space of this part of the bone is replaced by tumour tissue. × 40.

FIG. 20.—Invasion of muscle adjacent to bone by spindle-celled tumour tissue. From the tumour of the left femur in Rabbit 10. × 40.

FIG. 21.—An early metastasis in lung tissue. From Rabbit 5. × 40.

FIG. 22.—A metastasis in the liver. From Rabbit 11. × 50.

Barnes, Denz and Sissons.

7

8

9

10

11

12

Barnes, Denz and Sissons.

13

14

15

16

Barnes, Denz and Sissons.

17

18

19

20

21

22

Barnes, Denz and Sissons.

cartilaginous differentiation, but occasional regions of periosteal bone formation are present, and metaplastic cartilage produced by the displaced periosteum is included in some areas of tumour tissue.

Two small separate tumour nodules are present in the right scapula. One consists of spindle-celled fibrous tissue, while the other is a uniformly bony lesion, whose regular trabeculated pattern is shown in Fig. 19.

Each femur presented radiographic evidence of extensive medullary bone-formation. Histological examination revealed general fibrosis of the bone marrow, with scattered areas of bone-formation. Numerous bulky masses of beryllium-containing phagocytes were present in this tissue. In the lower part of each femur the marrow is completely replaced by a mass of bone-forming tumour tissue, which has begun to extend into the Haversian canals of the dense cortical bone. However, little erosion of cortical bone has been produced in this way, and tumour growth has not yet altered the external contour of the bones.

The tumour at the upper end of the right tibia is seen in Fig. 15, while Fig. 10 is the radiograph of the same lesion. This tumour consists partly of cartilaginous tissue (Fig. 17), partly of osteoid and bony tissue, and partly of undifferentiated spindle-celled tissue. The bone marrow in the lower part of the tibia is fibrosed, and contains areas of medullary bone formation. Aggregates of phagocytic cells loaded with silicate particles are present.

*Rabbit* 10.

At autopsy this animal presented a huge tumour of the left femur. Ossifying metastases were present in pelvic and abdominal lymph nodes, and in lungs and liver. Smaller tumours were found in the right humerus and the left tibia, and extreme medullary bone formation was present in the remaining long bones.

In radiographs the lesion of the left femur (Fig. 8) is densely ossified, and the greater part of the tumour tissue consists of calcified bone trabeculae. Histologically the tumour is a typical ossifying bone sarcoma, as illustrated in Fig. 18. Adjacent muscle is infiltrated by anaplastic spindle-celled tumour tissue (Fig. 20).

The tumour of the humerus is also a sclerosing one, consisting of anaplastic spindle-celled tissue with scattered areas of osteoid and bony differentiation.

The structure of the tumour of the left tibia is more varied. Some areas consist entirely of cartilaginous tissue, others show extensive osteoid and bony differentiation, while in some places the two tissues merge into a composite " osteochondroid " pattern. Undifferentiated spindle-celled tissue is also present. This varying histological structure is reflected in the radiographic appearance of the lesion, and in Fig. 11 areas of tumour bone formation and of calcification in cartilage can both be seen.

Sections of other long bones, where medullary bone formation had been identified radiologically, showed complete replacement of bone marrow by tumour tissue. In several situations this was beginning to invade the dense cortex of the bone concerned, and to expand the covering periosteum.

In this animal the body of one lumbar vertebra contained a small rounded area of calcifying spindle-celled tumour tissue, 2 mm. in diameter, sharply demarcated from the surrounding normal marrow. This was regarded as a metastasis rather than an independent primary tumour.

*Rabbit* 11.

At autopsy a tumour of the upper part of the right tibia was present. Histologically this is a typical ossifying bone sarcoma showing uniform and conspicuous bone formation. Ossifying metastases were present in the lungs.

The shaft of the tibia remote from the tumour shows the same fibrous replacement described in Rabbit 4. Again, there is extensive development of bone-trabeculae in the fibrous tissue. Here, however, all stages of transition between fibrous tissue, developing bone trabeculae and malignant bone-forming tumour tissue are present in the same lesion. This suggests that a continuous process is concerned in the development of frankly malignant tissue from the earlier pre-invasive lesion seen, for example, in the humerus of Rabbit 4.

In this animal other parts of the skeleton were not studied in detail.

*Rabbit* 13.

At autopsy a tumour of the upper part of the right tibia was present, but no metastases were found. Histologically the tumour shows marked structural variation, bony, cartilaginous and anaplastic undifferentiated tissue being present.

The bone marrow of the right femur shows numerous focal aggregates of macrophages loaded with beryllium silicate particles. Radiographs of this animal at the time of death showed early medullary bone formation in each humerus and in the left tibia and femur. These bones were not studied further.

### *Release of Beryllium in Tissues.*

Soluble salts of beryllium have been shown by Aldridge, Barnes and Denz (1949) to be extremely toxic. When injected subcutaneously the beryllium becomes fixed to tissue proteins and remains *in situ* to produce chronic granulomatous lesions.

The beryllium silicates are so insoluble, even in strong acids, that they cannot be brought into solution for estimation by ordinary chemical analysis. But chemical analysis shows that some free beryllium ions are present in aqueous suspensions of beryllium silicates. One mg. of insoluble beryllium silicate contains 5 $\mu$g. of soluble beryllium, and similarly 1 mg. of insoluble zinc beryllium silicate contains 130 $\mu$g. of soluble beryllium. Treatment of the refractory beryllium silicates with N/100 hydrochloric acid produces a 10 to 20-fold increase in the proportion of soluble beryllium in the aqueous suspension. In order to investigate the release of beryllium from refractory silicates a series of intradermal injections of 0·1 ml. of a 10 per cent. suspension of zinc beryllium silicate was given to a rabbit. At monthly intervals a piece of skin including the injection site was removed by biopsy and histological sections examined. A chronic inflammatory lesion develops around the silicate particles that have been taken up by macrophages. These lesions showed little resolution in the 7-month observation period. When stained by the method developed by Denz (1949) for the histochemical detection of beryllium these sections showed that some soluble beryllium had left the necrotic area and stained the surrounding fibrous tissue faintly and diffusely. This is evidence that soluble beryllium is liberated from the refractory beryllium silicates when in contact with tissues.

DISCUSSION.

These tumours are of interest because of their similarity to human bone sarcomas. Tumours of the same type have been produced in rabbits by radium (Ross, 1936). The range of structural variation in the human bone sarcomas and in those induced by beryllium in the rabbit is apparently identical. Among the experimental tumours some show extensive sclerosis and bone formation, others show cartilagincus differentiation, while a few are anaplastic masses with marked osteolytic propensities. But, just as in human tumours, it is not unusual for several or all of these different structural patterns to be found within a single lesion.

In one respect these tumours differ in their behaviour from their human counterparts. In addition to metastasing by the blood stream, local lymph-node deposits were found in 3 of the 4 animals with tumour dissemination. This frequency of lymph node metastasis is far higher than that occurring in human cases.

The material available from the animals in this experiment made it possible to trace the development of these tumous within the affected bones. While the fully developed tumours present a very different appearance from the medullary bone formation that has been described as an early stage of malignancy, all stages of transition between the two can be traced. It is suggested that these indicate a continuous process of transformation from the early medullary fibrosis to the later obviously malignant lesions, but this is only the interpretation of a number of morbid anatomical observations. Supporting this belief is the occurrence of malignant tissue within the medulla of long bones, while the cortex remains relatively unaffected.

The relationship between the small nodules which are the first change seen within the bone marrow and the subsequent fibrosis and malignant change is uncertain. The nodules produced by the silicates containing beryllium and those produced by the zinc silicate are indistinguishable by the usual histological methods. It seems clear, however, that it is the presence of the beryllium within the nodule that is in some way responsible for the progressive changes that lead eventually to malignancy. Although the beryllium silicates are considered to be insoluble, the observations on material injected intradermally into a rabbit showed that beryllium can be liberated from the particles and can diffuse into the surrounding tissues. Experience of the behaviour of both soluble and insoluble beryllium compounds leads to the conclusion that its toxic action is exerted locally at the site of inoculation or deposition.

Therefore, if beryllium is to be held directly responsible for the tumours, it is much more likely that this beryllium is liberated from deposits in the marrow rather than derived from more distant deposits. The possibility that the silicate may play some part in the development of the tumours is excluded by the fact that Gardner and Heslington (1946) produced tumours with beryllium oxide, and more recently Barnes (1950) has reported that two rabbits that received intravenous injections of a suspension of beryllium metal particles have developed characteristic sarcomas.

It is also apparent that the tissues themselves must contribute a factor, because similar nodules in the liver and lungs arouse very little fibrous tissue reaction, while in the spleen fibrosis and atrophy may go to extreme lengths without showing evidence of malignant change.

SUMMARY.

Bone sarcomata in rabbits developed after a course of intravenous injections of suspensions of zinc beryllium silicate and beryllium silicate, but not after zinc silicate.

Of 17 rabbits surviving the course of injections of zinc beryllium silicate 6 developed tumours ; of 11 survivors of the beryllium silicate injections 1 developed tumours.

Tumours were found between 39 and 83 weeks after the course of injections.

The bone tumours showed a great range of structural variation. The origin of the tumours was multicentric. Malignant blood-borne metastases were common.

The beryllium was considered to be causative. The injected materials were not radioactive.

Since this paper was submitted one further rabbit has died with a sarcoma of the pelvis 30 months after the last of a series of injections of zinc beryllium silicate.

The materials used in these experiments were supplied to us by the Research Laboratories of the General Electric Company, Wembley, to whom we would like to express our thanks.

REFERENCES.

ALDRIDGE, W. N., BARNES, J. M., AND DENZ, F. A.—(1949) *Brit. J. exp. Path.*, **30**, 375.
BARNES, J. M.—(1950) *Lancet*, i, 463.
CLOUDMAN, A. M., VINING, D., BARKULIS, S., AND NICKSON, J. J.—(1949) *Amer. J. Path.*, **25**, 810.
DENZ, F. A.—(1949) *Quart. J. micr. Sci.*, **90**, 317.
GARDNER, L. V., AND HESLINGTON, H. F.—(1946) *Fed. Proc.*, **5**, 221.
McKEAG, A. H., AND RANBY, P. W.—(1947) *Industr. Chem.*, **23**, 597.
NASH, P.—(1950) *Lancet*, i, 519.
ROSS, J. M.—(1936) *J. Path. Bact.*, **43**, 267.